# Routine vs. expert-guided transvaginal ultrasound in the diagnosis of endometriosis: A retrospective review

**DOI:** 10.1007/s00261-014-0243-5

**Published:** 2014-09-19

**Authors:** Margaret Ann Fraser, Sugandha Agarwal, Innie Chen, Sukhbir Sony Singh

**Affiliations:** 1 Department of Medical Imaging, University of Ottawa, 501, Smyth Road, Ottawa, ON K1H8L6 Canada; 2 Clinical Epidemiology Program, Ottawa Hospital Research Institute, Ottawa, ON K1Y4E9 Canada; 3 Department of Obstetrics and Gynecology, University of Ottawa, Ottawa, ON K1H7W9 Canada; 4 Ottawa Hospital Research Institute, Ottawa, ON K1Y4E9 Canada; 5 Chronic Disease Program, Ottawa Hospital Research Institute, Ottawa, ON K1Y4E9 Canada

**Keywords:** Diagnosis, Endometriosis, Imaging, Transvaginal ultrasound

## Abstract

**Objective:**

The objective of this study is to evaluate the sensitivity of routine trans vaginal ultrasound (TVUS) compared to expert-guided transvaginal ultrasound (ETVUS) for the diagnosis of endometriosis.

**Methods:**

A retrospective chart review performed at a Canadian tertiary center specializing in the diagnosis and management of endometriosis. All cases with surgically confirmed endometriosis and an ETVUS completed at a single center were included for review and compared to routine TVUS performed for the same indication.

**Results:**

Forty cases met the inclusion criteria. Mean patient age of the study population at first surgical diagnosis was 31.2 ± 6.9 years. Dysmenorrhea (76.9 %) and chronic pelvic pain (74.3 %) were the most common presenting symptoms. Sensitivity of routine TVUS was 25 % (10/40), compared to 78 % (31/40) with ETVUS, (*P* < 0.01). Comparisons were made based on site of disease. Routine TVUS and ETVUS detected bladder involvement in (0/40) vs. 5 % (2/40); ureter (0/40) vs. 7.5 % (3/40); ovary 25 % (10/40) vs. 72.5 % (29/40); retrocervical area (0/40) vs. 60 % (24/40), rectosigmoid 5 % (2/40) vs. 77.5 % (31/40), respectively. Specific endometriotic lesions recognized by TVUS versus ETVUS, were: ovarian endometriomas in 25 % (10/40) vs. 45 % (18/40), adhesions leading to abnormal anatomy in 2.5 % (1/40) vs. 77.5 % (31/40); endometriotic implants or plaques in 2.5 % (1/40) vs. 70 % (28/40); and endometriotic nodules in 2.5 % (1/40) vs. 35 % (14/40), respectively. Routine TVUS diagnosis relied on the presence or absence of endometrioma (10/10), whereas ETVUS showed additional sites of disease in 97 % (30/31) patients.

**Conclusions:**

ETVUS is more sensitive than routine TVUS to diagnose endometriosis, identifying lesions other than endometrioma and is of assistance in surgical planning and patient counseling.

Endometriosis is defined as the presence of endometrium-like tissue outside the uterus. It is a chronic and often progressive condition estimated to affect 5 %–10 % of reproductive aged women [1, 2]. Despite such high prevalence, endometriosis remains an enigmatic disease with a poorly understood pathophysiology. Symptoms of dysmenorrhea, dyspareunia, dyschezia, dysuria, chronic pelvic pain, and infertility associated with endometriosis are often highly debilitating and have a major negative impact on women’s sexual life, self-esteem, and quality of life [3, 4].

Although endometriosis is common and thus has a significant societal burden, the average diagnostic delay from onset of symptoms is often from 6 to 10 years [1, 5]. Therefore, it is essential to consider efforts that may decrease diagnostic delays and help guide patients to the appropriate healthcare providers and resources. Earlier diagnosis of endometriosis should be combined with accurate evaluation of extent of disease, which may in turn help prepare for surgical intervention when required. Accurate preoperative evaluation may help reduce the number of incomplete or failed surgeries due to inadequate knowledge of the extent and severity of disease and also direct referrals to specialized centers or specialists if needed.

Transvaginal ultrasound (TVUS) is the recommended first-line imaging modality for endometriosis [6]; however, limitations of this approach have been well documented in the literature [7–9]. An expert-guided transvaginal ultrasound (ETVUS) has been shown to improve detection rates of endometriosis and provide a preoperative assessment of the extent of disease, especially in cases with deep disease [10–13]. Recent ESHRE guidelines [14] also reiterate the role of TVUS performed by experienced clinicians in the diagnosis of endometriosis other than ovarian forms.

The aim of this study was to evaluate the sensitivity of routine trans vaginal ultrasound (TVUS) compared to ETVUS for the diagnosis of endometriosis in a tertiary level Canadian center.

## Materials and methods

This retrospective analysis was performed at a Canadian tertiary center specializing in diagnosis and management of endometriosis. The Ottawa Health Science Network Research Ethics Board (OHSN-REB) approved this study.

### Review strategy

**Fig. 1:**
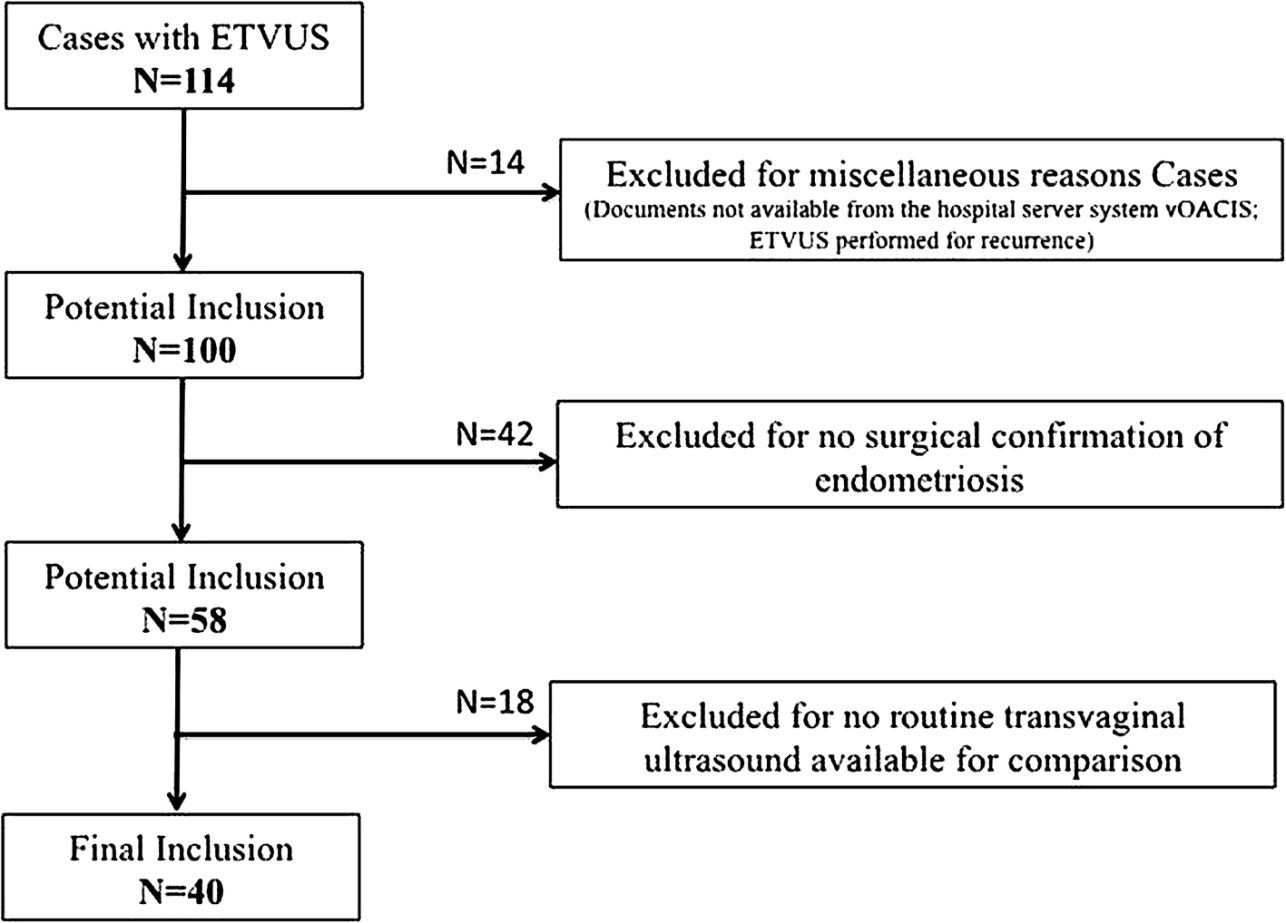
Review strategy.

A total of 114 women between 2006 and 2013 who underwent an ultrasound for pelvic pain by a single expert radiologist (MF) were identified. A single reviewer reviewed electronic case files of all the identified cases to determine inclusion in our study. Cases were included if they had at least one surgical confirmation of endometriosis and also had had a routine TVUS performed for the same indication (Fig. 1). Forty women met the inclusion criteria and were included in the final analysis. A standardized intake sheet was used to include details about clinical history, physical examination, prior surgical or medical management, and findings from routine TVUS as well as ETVUS to extract the necessary data points from the study group.

### Imaging modalities

#### Routine transvaginal ultrasound (TVUS)

Routine TVUS was defined as a pelvic scan that was performed by a sonographer who recorded the static images and video clips, later read by a radiologist or a gynecologist. The information on the type of ultrasound machine used was not accessible as it varied greatly by location of scans done. However, all reports must have stated a “satisfactory” quality of imaging to allow appropriate reporting. Ultrasounds done either at the same center or performed outside with reports made available to the center’s system were reviewed.

#### Expert guided transvaginal ultrasound

ETVUS was defined as a real time, hands-on dynamic ultrasound performed by an expert clinician with experience in performing a focused endometriosis scan. In our study, this was a single radiologist (MF) with a developed practice in ultrasound for pelvic pain and endometriosis. The ultrasound was performed using a Philips IU22, 2006 model with a C8-4 endocavity probe (Philips Healthcare, Andover, MA, USA).

Prior to performing the ultrasound, the radiologist undertook a relevant clinical history pertaining to symptoms related to endometriosis. Each patient underwent a methodical ultrasound examination in the following order:

- Complete suprapubic pelvic ultrasound to assess uterine as well as ovarian position, size and morphology; appearance of cul-de-sac; presence of pelvic masses.
- Limited abdominal ultrasound of the kidneys to document renal size and morphology (i.e. evaluation for hydroureteronephrosis).
- Compartment based reporting of endometriotic lesions that included an anterior, middle, and posterior heading.

**Fig. 2:**
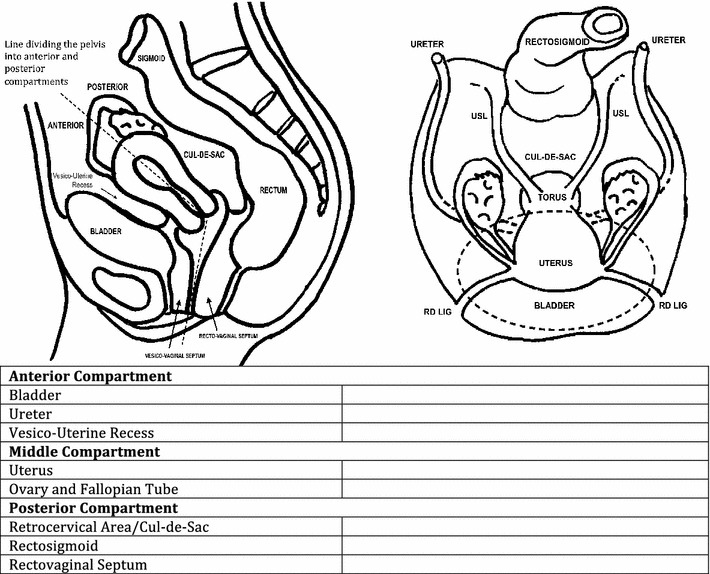
Template used to report expert transvaginal ultrasound.

The anterior compartment included lesions involving bladder and ureter; middle compartment included lesions involving uterus, ovary, and fallopian tubes; posterior compartment was divided into three major areas according to involvement—*retrocervical* area (lesions involving uterine torus, pouch of douglas, proximal uterosacral ligaments, posterior vaginal fornix, and posterior uterine serosa), *rectosigmoid* (lesions involving rectum, sigmoid, rectosigmoid junction), and lesions involving *rectovaginal septum*. The impression provided in the report was used for a positive or negative diagnosis of endometriosis (Fig. 2).

### Data collection

Reports from routine TVUS and ETVUS were considered satisfactory for comparison when the scan was performed for pelvic pain in each case, and a structured report with reasonable evaluation of uterus and bilateral ovaries was available. The diagnosis of endometriosis was based on the impression provided in the report. Only cases where a specific diagnosis of endometriosis was provided were considered as positive, whereas reports without a definitive diagnosis or suggestion of endometriosis were considered as negative.

### Statistical analysis

Characteristics of the study sample were described using frequencies and proportions for categorical variables and means and standard deviations for continuous variables. To compare the sensitivities of the two modalities in the diagnosis of endometriosis, McNemar’s Chi-squared test was used, and 95 % confidence intervals (CIs) for sensitivity were provided as well. Statistical significance was set at an alpha level of 0.05. All analyses were performed using SAS-PC statistical software version 9.3 (SAS Inc., Cary, NC).

## Results

### Patient data

A total of forty surgically confirmed cases of endometriosis were included in the analysis. The age of women at first surgical diagnosis ranged from 22 to 48 years with a mean of 31.2 ± 6.9. As shown in Table 1, dysmenorrhea (76.9 %) and chronic pelvic pain (74.3 %) were the most common presenting symptoms. Significant number of women had additional menstrual, bowel, and bladder symptoms in the form of abnormal uterine bleeding, per rectal bleeding, constipation, bloating, and irritative lower urinary tract symptoms.

**Table 1:** Patient characteristics

Characteristics	Number of patients (%) *N* = 40 (unless specified)
Mean age (years) (range)	31.2 ± 6.9 (22–48)
Mean gravidity	0.8 ± 1.3
Nulliparous women	20 (51.2)^a^
Desirous of fertility	20 (55.5)^b^
Symptoms^a^	
Dysmenorrhea	30 (76.9)
Dyspareunia	21 (53.8)
Dysuria	7 (17.9)
Dyschezia	21 (53.8)
Chronic pelvic pain	29 (74.3)
Other symptoms^c^	
Abnormal uterine bleeding	16 (40)
Bowel symptoms	12 (30)
Urinary symptoms	5 (12.5)
Hormonal treatment^d^	27 (67.5)

Bowel symptoms (per rectal bleeding, bloating, constipation, and intermittent bowel obstruction)

Urinary symptoms (hematuria, frequency, urgency)

^a^Data available for 39 cases

^b^Data available for 36 cases

^c^Abnormal Uterine Bleeding (heavy menstrual bleeding, irregular heavy menstrual bleeding, and extended vaginal spotting)

^d^Hormonal treatment included combined oral contraceptive pills, GnRH analogs, hormonal intrauterine devices, progesterone like including dienogest, and depot medroxyprogesterone acetate

### Routine TVUS findings

**Fig. 3:**
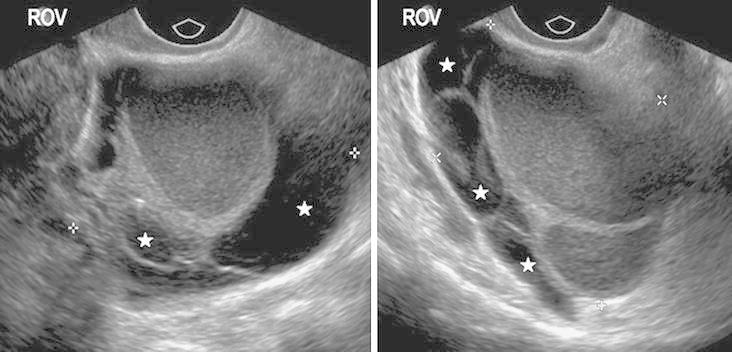
Transvaginal ultrasound images showing typical features of a right ovarian endometrioma (ROV): multiloculated cystic structure, uniform low-level internal echoes with peripheral “candle wax” appearance due to repeated hemorrhage of variable age (*filled star*).

Routine pelvic ultrasound provided a diagnosis of endometriosis in 10/40 (25 %) patients (Table 2). In five other patients, no definitive diagnosis of endometriosis was provided and was included as a differential or a probable diagnosis. Ovary was found to be the most commonly involved site with ovarian endometrioma as the most common lesion detected in 10/40 (25 %) women (Fig. 3). Bowel involvement, identified with presence of nodule, implant or adhesions was detected in two cases. Endometriotic disease in retrocervical area (including posterior vaginal fornix, uterosacral ligaments, and pouch of douglas), rectovaginal septum, bladder, and ureter was not identified in any of the patients.

**Table 2:** Routine transvaginal ultrasound compared to expert-guided transvaginal ultrasound in the diagnosis of endometriosis

Modality	*N* (*N* = 40)	Sensitivity (%)	95 % CI	*P* value
Routine transvaginal ultrasound (TVUS)	10	25	12.71–41.20	<0.01
Expert-guided transvaginal ultrasound (ETVUS)	31	77.50	61.54–89.14

### ETVUS findings

The expert TVUS suggested the diagnosis of endometriosis in 31/40 (77.5 %) women. Probability of endometriosis was suggested in two other patients due to likely appearance of fixed pelvic small bowel and a plaque-like hypoechoic thickening at the anterior margin of cecum (1), plaque-like material on uterine serosal margin (1) but no definitive diagnosis of endometriosis was provided.

Review of the anterior compartment findings revealed endometriosis involving the bladder in 2/40 (5 %) of women and ureteric involvement in 3/40 (7.5 %) women (Table 3). All the cases of ureteric involvement were extrinsic, and two out of three were associated with ipsilateral hydroureteronephrosis. One woman showed presence of implants in the vesicouterine recess. The anterior compartment, therefore, was overall involved in 6/40 (15 %) women as detected by ETVUS.

**Table 3:** Compartment wise distribution of endometriotic lesions as imaged by routine transvaginal ultrasound compared to expert-guided transvaginal ultrasound

Location of endometriotic lesion	Routine transvaginal ultrasound *N* (%) (*N* = 40)	Expert-guided transvaginal ultrasound *N* (%) (*N* = 40)
Anterior compartment	0	6 (15)
Urinary bladder	0	2 (5)
Ureter	0	3 (7.5)
Vesicouterine recess	0	1 (2.5)
Middle compartment	10 (25)	29 (72.5)
Uterus	0	14 (35)
Ovary and fallopian tube	10 (25)	29 (72.5)
Posterior compartment	2 (5)	31 (77.5)
Retrocervical area	0	24 (60)
Uterosacral ligaments	0	12 (30)
Obliterated pouch of douglas	0	16 (40)
Posterior vaginal fornix	0	6 (15)
Posterior uterine serosal margins	0	6 (15)
Uterine torus	0	7 (17.5)
Rectosigmoid	2 (5)	31 (77.5)
Rectovaginal septum	0	0

Ovarian involvement in the middle compartment was reported in 29/40 (72.5 %) women. Adhesions involving the adnexa was the most common lesion, found in 28/40 (70 %) cases, whereas endometriomas were detected in 18/40 (45 %) patients. Retroflexed uterus usually considered as a sign of endometriosis was detected in 13/36 (36.1 %) (four patients were post hysterectomy). The middle compartment was involved in 29/40 (72.5 %) cases, diagnosed by ETVUS.

ETVUS diagnosed disease involving the retrocervical area in 24/40 (60 %) women with adhesions in pouch of douglas leading to frozen appearance in 16/40 (40 %), uterosacral ligament disease in 12/40 (30 %), extension of disease in the posterior vaginal fornix as well as posterior uterine serosal margins in 6/40 (15 %) women, in decreasing order. Rectosigmoid (including lesions of sigmoid, rectum, and rectosigmoid junction) was found to be the most common site of disease involved in 31/40 (77.5 %) of the patients.

None of the women showed disease of the rectovaginal septum. Posterior compartment was found to be the most commonly involved compartment with disease in 33/40 (82.5 %) women.

**Fig. 4:**
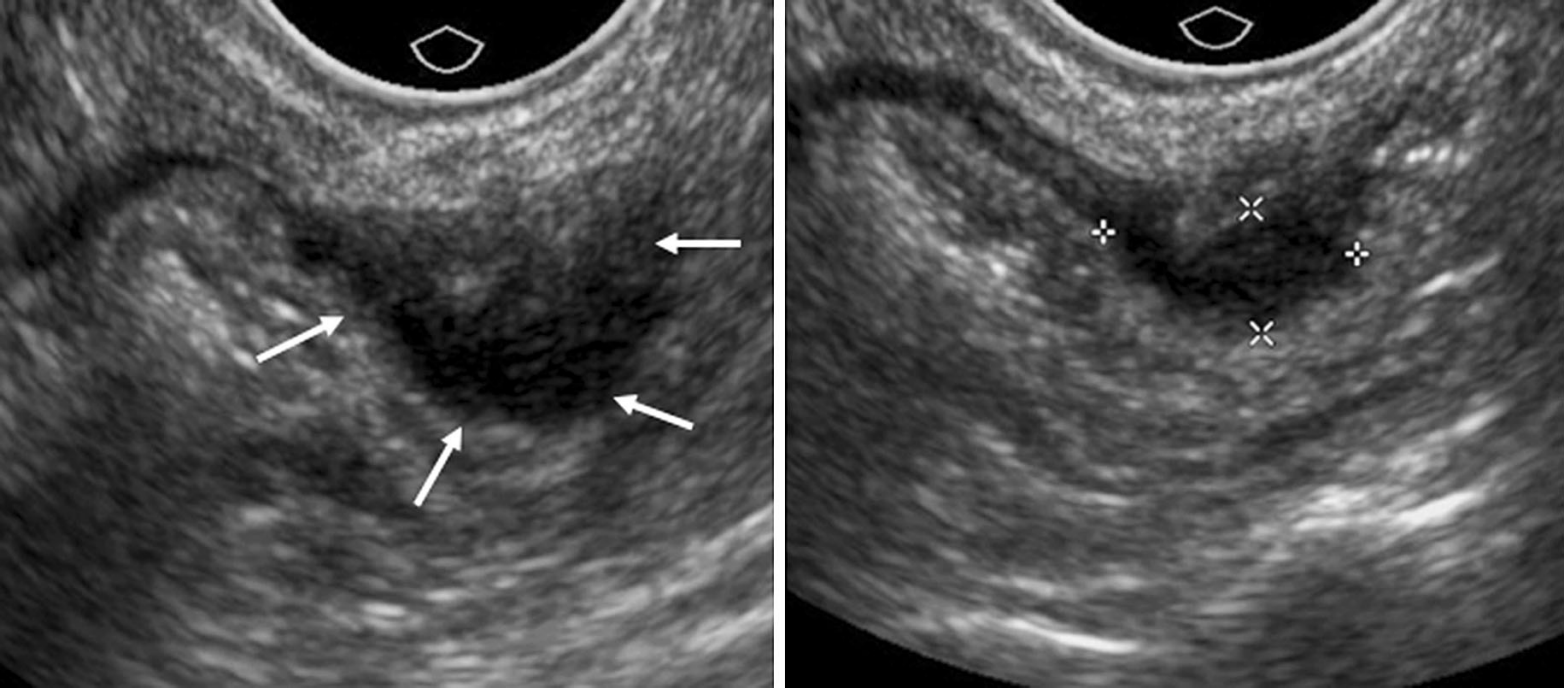
Expert transvaginal ultrasound (ETVUS) images showing endometriosis involving the anterior rectum, appearing as a solid, non-compressible, hypoechoic implant (*white arrows*, electronic calipers). Unlike routine transvaginal ultrasound, ETVU assesses the rectum and sigmoid for improved sensitivity.

Other than ovarian endometriomas, ETVUS identified lesions such as endometriotic nodule (defined as a lesion ≤20 mm, rounded or lobulated in appearance); endometriotic implant or plaque (Fig. 4) (defined as an oblongated lesion occurring at surface usually at bowel, retrocervical area, or uterine serosa); adhesions leading to abnormal pelvic anatomy; and endometriotic mass (defined as a lesion >30 mm, confluent in nature involving one or more organs) (Table 4). Adhesions involving pelvic structures and causing fixation, tethering, or kinking effect was found to be the most common lesion occurring in 31 (77.5 %) women followed by endometriotic implants/plaques in 28/40 (70 %) women. Also noteworthy was that isolated lesions were very uncommon occurring in only three women, and endometriotic involvement was multifocal in most of the cases. The dynamic nature of the modality was also able to identify foci of tenderness replicating the patient’s pain in 17/40 (42.5 %) of women.

**Table 4:** Comparison of different type of endometriotic lesions identified with routine transvaginal ultrasound and expert-guided transvaginal ultrasound

Lesion	Routine transvaginal ultrasound *N* (%) (*N* = 40)	Expert-guided transvaginal ultrasound *N* (%) (*N* = 40)
Ovarian endometrioma	10 (25)	18 (45)
Adhesions causing abnormal anatomy (fixation, tethering, kinking effect)	1 (2.5)	31 (77.5)
Endometriotic nodule	1 (2.5)	14 (35)
Endometriotic plaque/implant	1 (2.5)	28 (70)
Endometriotic mass	0	3 (7.5)
Focus of tenderness replicating patient’s pain	0	17 (42.5)

## Discussion

This series conducted at a Canadian tertiary care center for endometriosis confirmed that an advanced TVUS performed by an expert clinician trained in focused endometriosis scanning is likely to be more advantageous (sensitivity 77.5 %) in the diagnosis of endometriosis, especially for sites of involvement other than ovary, when compared to routine pelvic ultrasound performed by a sonographer (sensitivity 25 %).

Similar low sensitivity in detecting endometriosis by routine pelvic ultrasound has been shown by earlier studies. Friedman et al. in their review of 37 women assessed for infertility depicted successful detection of endometriosis in only 10.8 % (4/37) with routine ultrasound, concluding that it is neither sensitive nor specific in diagnosing endometriosis [13]. It is important to note that the focus with routine ultrasound is on the disease’s presentation as a discrete pelvic mass, mostly endometriomas [6–8], and not on other forms of endometriosis, a fact that was replicated in this retrospective review. The diagnosis of endometriosis in all the ten women was based exclusively on the presence or absence of endometrioma with features other than endometriomas identified in only two women. TVUS, otherwise, is considered a useful test both to make and to exclude the diagnosis of an ovarian endometrioma, as concluded by a systematic review assessing the use of gray-scale imaging in the diagnosis of ovarian endometriomas specifically rather than the full spectrum of endometriosis lesions [14]. A definitive advantage of routine TVUS over ETVUS remains its easy availability, cost and time efficacy as well as no requirement for additional, specialized training.

**Fig. 5:**
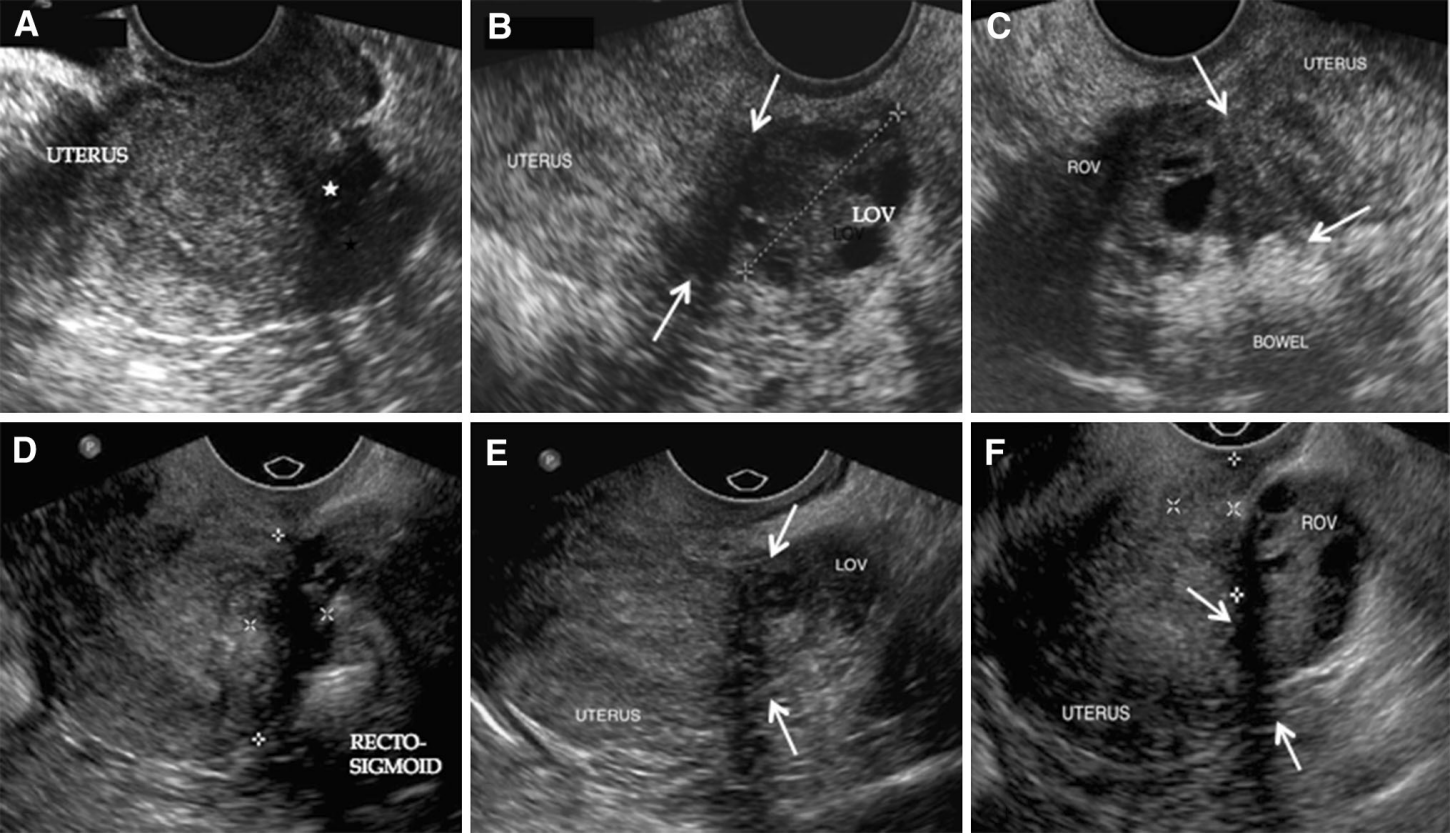
Routine (**A**–**C**) and expert (**D**–**F**) transvaginal ultrasound images of endometriosis. Although routine transvaginal ultrasound depicts typical features of endometriosis, lack of familiarity with and failure to report findings other than endometrioma lead to poor sensitivity. **A** Routine ultrasound image depicting typical hypoechoic endometriotic tissue causing fixed uterine retroversion and obliteration of the cul-de-sac (*filled star*). **B** Hypoechoic endometriotic implants between the left ovary (LOV) and the uterine fundus (*white arrows*). **C** Adherence of bowel to the right ovary (ROV). Routine transvaginal ultrasound diagnosis was normal. Expert transvaginal ultrasound images in the same case depict similar findings, but recognition and diagnosis lead to improved sensitivity. **D** Solid hypoechoic endometriotic plaque causing fixed uterine retroversion, obliterating the cul-de-sac and adhering adjacent tissues (electronic calipers). **E** Hypoechoic endometriotic implant causing left ovarian (LOV) fixation to the uterine fundus. **F** Right ovary (ROV) adherent to uterine fundus (*white arrows*). Endometriotic tissue fills the retrocervical space (electronic calipers).

A good quality TVUS when performed by an expert has shown a high degree of accuracy in the diagnosis of endometriosis [9–11]. The sensitivity of 77.5 % achieved in the current review is comparable to 78.5 % reported by Bazot et al. in their study assessing the diagnostic accuracy of TVUS in deep endometriosis [15]. The scan when performed by an experienced radiologist is highly sensitive in detecting endometriosis of the pelvis not only involving the ovaries but also structures such as the vagina, retrocervical space, uterosacral ligaments, bladder, and rectal wall [9, 15–20]. Two essential components of ETVUS are a detailed compartmentalized assessment of the disease to identify the extent as well as severity of involvement, and detection of different types of lesions pertaining to the deep involvement of the disease such as nodules, dense adhesions, implants, and obliteration of the Pouch of Douglas. Both of these factors are critical for symptomatic evaluation and formulating a management plan. Figure 5 describes a case where although TVUS depicts typical features of endometriosis but are not reported due to lack of familiarity of the lesions. Expert TVUS images in the same case depict similar findings missed on TVUS.

Imaging of the urinary tract to identify endometriotic disease is important [19]. Disease may be easily overlooked with the TVUS as urinary tract imaging does not form a part of the standard protocol used for pelvic pain. ETVUS, on the other hand with its detailed compartmentalized assessment, is more likely to pick up the disease. Three women in this series, missed by TVUS, were taken up for surgical removal of the disease. ETVUS appears a useful tool in experienced hands but its performance in the absence of clinical suspicion or a negative clinical exam, requirement of specific training to understand the radiological features, and limited availability are considerable limitations.

The major implications of missed diagnosis especially with a nonexpert scan are delay in diagnosis, rationalizing patient’s symptoms as normal, delay in referral to a specialized center, inappropriate management, and incomplete surgeries amounting to patient dissatisfaction. A total of 68 surgeries were performed for the management of endometriosis in this study group of forty, averaging 1.7 per patient. Twenty-seven women (27/40, 67.5 %) in this review attended a specialized center for further management after being treated at other non-specialized centers. All of these women had undergone prior surgery, with incomplete surgical management or continued signs and symptoms. Sixteen out of these 27 (16/27, 59.2 %) women underwent a repeat definitive surgery after being evaluated by an expert scan at the specialized center. The duration between primary management at other centers and attendance at the specialized center ranged from less than 1 year to as long as 13 years. These unplanned or incomplete surgeries as well as long diagnostic delays can add to economic burden on the healthcare system emphasizing the need for an accurate and early preoperative diagnosis of the disease.

The results show that there should be no argument against a high quality ultrasound; however, it is difficult to completely eliminate the inherent biases in the comparative study as this one. The significant ones being that the TVUS was not standardized for technique, time period, ultrasound machine, or the imager’s experience. It is extremely difficult to standardize these factors as the TVUS was performed at different locations; however, to minimize this bias, we ensured that satisfactory quality of imaging with a reasonable evaluation of uterus and bilateral ovaries was available. It is also difficult to estimate the extent of influence on ETVUS when performed after the prior information of TVUS results. Considering that endometriosis has a chronic progression over years, the median time difference of 294 days between TVUS and ETVUS seems reasonable, though, it is hard to be certain if the higher detection rate with ETVUS pertains to progression of disease or missed diagnosis with TVUS. In the authors' experience, it would be highly unlikely that 30 out of 40 women had a progressive endometriosis developing within a relatively short-time period. The reason it is so important to share this knowledge is the fact that 30 out of 40 scans in patients with surgically confirmed endometriosis-related pelvic pain were reported as “normal”. It is essential to share with our colleagues the fact that a “normal” routine ultrasound may not be enough in patients who are suffering with severe pain.

In light of the above-discussed facts, it would be reasonable to state that routine pelvic ultrasound is a valuable first line of investigation for patients with pelvic pain, but it can neither reliably diagnose nor convincingly rule out the presence of endometriosis, especially the nonovarian form. The future emphasis, thus, should be toward developing TVUS imaging predictors other than endometriomas that are more reliable, easily identifiable, reproducible, and require a short learning curve with minimal training. There is a need to develop and disseminate the concept of a standardized level I screening ultrasound for endometriosis (that includes predictors other than endometriomas) to help primary healthcare providers in making a definitive diagnosis without delay as well as to assess the need for referral to a specialized centre, and a level II expert ultrasound for endometriosis, intended towards knowing the extent and severity of disease to help in surgical planning.

## Conclusions

Expert-guided TVUS is more sensitive than routine pelvic ultrasound in the diagnosis of endometriosis, especially the non-ovarian form. It also provides a detailed compartmentalized description of the extent and severity of the disease that may assist in surgical planning and patient counseling.

## Abbreviations

TVUS: Trans vaginal ultrasound
ETVUS: Expert-guided trans vaginal ultrasound
